# Screening for atrial fibrillation: a call for evidence

**DOI:** 10.1093/eurheartj/ehz834

**Published:** 2019-12-07

**Authors:** Nicholas R Jones, Clare J Taylor, F D Richard Hobbs, Louise Bowman, Barbara Casadei

**Affiliations:** 1 Nuffield Department of Primary Care Health Sciences, University of Oxford, Radcliffe Primary Care Building, Woodstock Road, Oxford OX2 6GG, UK; 2 MRC Population Health Research Unit, Nuffield Department of Population Health, University of Oxford, Old Road Campus, Oxford OX3 7LF, UK; 3 Radcliffe Department of Medicine, University of Oxford, Level 6 West Wing, John Radcliffe Hospital, Oxford OX3 9DU, UK

**Keywords:** Atrial fibrillation, Screening, Stroke, Anticoagulation

## Abstract

Atrial fibrillation (AF) is the most common cardiac arrhythmia and prevalence is predicted to double over the next 30 years due to changing demographics and the rise in prevalence of risk factors such as hypertension and diabetes. Atrial fibrillation is associated with a five-fold increased stroke risk, but anticoagulation in eligible patients can reduce this risk by around 65%. Many people with AF currently go undetected and therefore untreated, either because they are asymptomatic or because they have paroxysmal AF. Screening has been suggested as one approach to increase AF detection rates and reduce the incidence of ischaemic stroke by earlier initiation of anticoagulation therapy. However, international taskforces currently recommend against screening, citing the cost implications and uncertainty over the benefits of a systematic screening programme compared to usual care. A number of large randomized controlled trials have commenced to determine the cost-effectiveness and clinical benefit of screening using a range of devices and across different populations. The recent AppleWatch study demonstrates how advances in technology are providing the public with self-screening devices that are increasingly affordable and accessible. Health care professionals should be aware of the implications of these emerging data for diagnostic pathways and treatment. This review provides an overview of the gaps in the current evidence and a summary of the arguments for and against screening.

**Figure:**
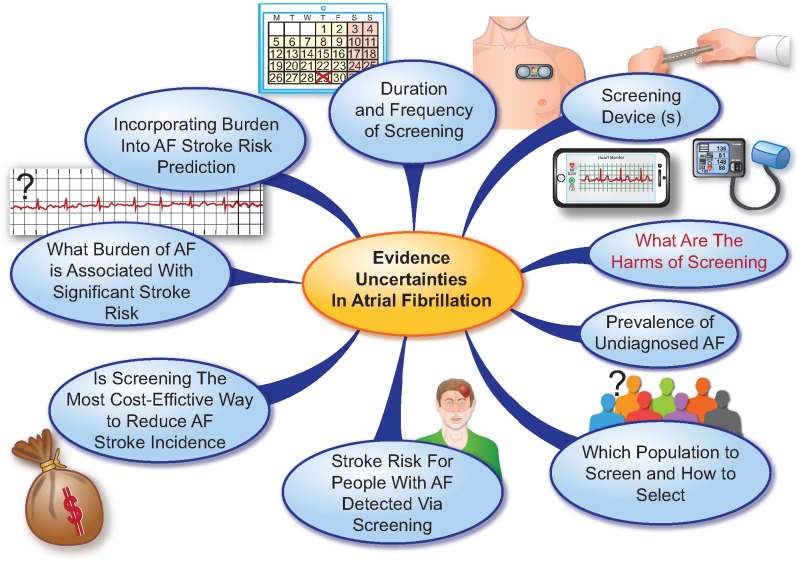

## Introduction

Atrial fibrillation (AF) is a public health and economic issue of epidemic proportion. In 2010, there were an estimated 33 million people worldwide with diagnosed AF and this figure is expected to double by 2050.^1^ The cost of caring for people with AF and its complications accounts for around 2% of total health care expenditure in high-income countries.^2^ AF is associated with substantial morbidity and mortality, largely related to an increase in the risk of cardiovascular disease, including a five-fold increased stroke risk.^3^^,^^4^

When used appropriately, oral anticoagulation is estimated to reduce stroke risk for people with AF by about 65% compared to placebo.^4^^,^^5^ For people with undiagnosed AF, ischaemic stroke may be the first clinical manifestation of the condition. Currently, 10% of people who have an ischaemic stroke are first diagnosed with AF at the time of the event.^6^ If it were possible to detect those who had asymptomatic AF earlier, it may be possible to prevent some of these strokes by offering anticoagulation.

Screening is suggested as one strategy to increase AF detection rates and start anticoagulation early in high-risk individuals. The emergence of direct oral anticoagulants and new technology potentially offering more accurate and varied approaches to AF diagnosis has resulted in renewed enthusiasm for screening in some quarters.^7^^,^^8^ Screening by opportunistic pulse palpation or electrocardiogram (ECG) rhythm strip is already recommended by the European Society of Cardiology (ESC) in all patients ≥65 years contacting health services and by the National Institute for Health and Care Excellence (NICE) where patients have a symptom suggestive of AF,^9^^,^^10^ based on the 60% improvement in AF detection compared to routine care over 12 months in the landmark SAFE trial.^11^ Yet, international taskforces continue to recommend against implementing systematic population-level screening in asymptomatic patients, citing the cost implications and a lack of evidence that it is more effective than usual care.^12^^,^^13^ A number of large randomized controlled trials (RCTs) have recently been funded to help determine if screening is cost-effective and improves patient prognosis.^14–16^ What are the knowledge gaps that these trials seek to address and will the results end the debate around the relative merits of AF screening?

## How many people currently have undiagnosed atrial fibrillation?

The rationale for screening is based on the supposition that there are a large number of people with undiagnosed AF and a stroke risk that warrants anticoagulation. Recent estimates suggest 15% of people with AF are currently undiagnosed, of whom up to 75% may be eligible for anticoagulation.^17^ A systematic review of AF screening amongst the general population with single time-point pulse palpation or ECG, found the incidence of previously undiagnosed AF was 1.4% in adults aged ≥65 years.^18^

Similar detection rates have been reported in recent trials of opportunistic screening. For example, when electronic reminders and decision support software were used to support opportunistic screening with the AliveCor KardiaMobile smartphone in primary care amongst all attending patients aged 65 years and older, 1805 of 11 476 (16%) of eligible patients were screened and 19 new cases of AF detected (1.1%).^19^ In another study, 184 Canadian general practitioners were asked to screen all the patients aged 65 years or older over a 3-month period, again using the AliveCor KardiaMobile. New AF was detected in 471 of the 7585 patients screened (6.2%). Limited information is given with regard to the study participants but it is suggested clinicians were targeting the screening at high-risk individuals, which would explain the higher reported prevalence.^20^

Relying on opportunistic detection of AF using single time-point pulse palpation or ECGs will miss cases where people are oligo- or asymptomatic and those with paroxysmal AF who are in sinus rhythm at the time of assessment.^21^ For example, the Swedish STROKESTOP Study screened 7173 participants aged 75 or 76 years from the general population with a single ECG and found 0.5% had previously undiagnosed AF.^22^ This rose to 3% with repeat serial ECGs over a 2-week period—above a four-fold increase in detection.^22^

**Figure 1:**
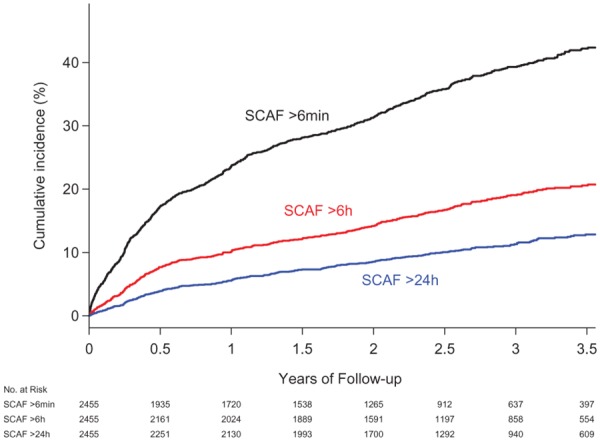
Kaplan–Meier estimates of cumulative incidence of subclinical atrial fibrillation (SCAF) >6 min, >6 h, and >24 h. Reproduced from the Van Gelder *et al*.^21^

Prolonged continuous ECG monitoring with either surface ECGs or implantable cardiac devices has detected new episodes of asymptomatic AF in up to 50% of subjects, depending on the type and duration of monitoring, patient characteristics, and the definition of AF (*Table 1*). The ASSERT study included 2455 participants aged >65 years with hypertension but no prior history of AF who were receiving a dual-chamber pacemaker or internal cardioverter-defibrillator.^21^ Over a mean 2.5-year follow-up, 18.8% of people developed asymptomatic AF. Many had short episodes of arrhythmia but 11% of participants had an episode of AF lasting over 24 h by 3-year follow-up (*Figure 1*).^21^

**Table 1:** Rates of atrial high rate episodes (AHRE) in patients with implanted cardiac devices (table modified from AF-Screen paper)^7^

Trial	Year	Device	Patient profile	Mean CHA_2_DS_2-_VASC	Median follow-up (years)	AF burden threshold	Incidence of AF	Adverse events
Ancillary MOST^23^	2003	PPMs	All patients eligible where PPM used for sinus node disease. 60% had history of SVT.	Not included in study	2.25	>5 min	50% (156/312)	Death or non-fatal stroke in 33/1160 (20.6%) of those with AF compared to 16/152 (10.5%) of those without.
TRENDS^24^	2012	PPMs and ICDs	No prior stroke or AF. ≥1 stroke risk factor.	≥2 in 70%	1.1 ± 0.7	>5 min	30% (416/1368)	Compared to no AF, adjusted hazard ratio for stroke, TIA, or systemic embolism in the high burden group was 2.20 (0.96–5.05) and in the low burden group 0.98 (0.34–2.82).^a^
ASSERT^25^	2012	PPMs and ICDs	History of hypertension without prior AF.	2.3 ± 1.0 for no AHRE. 2.2 ± 1.1 for AHRE detected	2.5	>6 min	34.7% (895/2580), including 10.1% at 3-month follow-up (261/2580)	No difference detected in ischaemic stroke or systemic embolism rate between those with or without AF.
IMPACT^26^	2015	ICDs and CRTDs	No prior AF, median age 64.4, 71.5% had ischaemic heart disease.	2 (median)	1.9	>4–12 s	34.8% (945/2718)	No difference in stroke, systemic embolism or major bleed between group treated with anticoagulation for new AF or control.
ASSERT II^27^	2017	ICM	No prior AF, age ≥65 years, CHA_2_DS_2_VASc ≥2, OSA, or a BMI >30 kg/m^2^ and either left atrial enlargement or elevated NT-proBNP ≥290 pg/mL.	4.1 ± 1.4	1.35 ± 0.3	>5 min	34.5% per patient year (95% CI 27.7–42.3). In total 90/256 detected with AF	Eight deaths, four ischaemic strokes—all in people without AF. One patient started anticoagulation for AF had a haemorrhagic stroke.

AF, atrial fibrillation; AHRE, atrial high rate episode; CRTD, cardiac resynchronization therapy device; ICD, implantable-cardioverter defibrillator; ICM, implantable cardiac monitor; PPM, permanent pacemaker.

a The cut-off threshold between high and low burden groups was based on the observed median AF burden amongst all people with AF.

A 2016 Cochrane Review on ‘Systematic screening for the detection of atrial fibrillation’ found only one study that met their inclusion criteria. This was the SAFE trial, a cluster randomized trial in UK primary care comparing routine care to either opportunistic pulse palpation or systematic screening using a single time-point ECG.^11^ Based on these results, the Cochrane review concludes that both opportunistic pulse palpation and systematic screening will increase AF detection, a position endorsed by the European Heart Rhythm Association.^28^ European Heart Rhythm Association has also previously highlighted the potential benefit of targeting screening in high-risk populations and suggest systematic ECG screening can be considered in people aged 75 years or older, or those at high stroke risk.^29^

The prevalence of asymptomatic AF detected will depend on the population screened, the device and the duration of monitoring. Would the additional cases identified via screening benefit from anticoagulation to the same extent as people with symptomatic AF?

## What is the stroke risk in people with asymptomatic atrial fibrillation?

A recent meta-analysis that compared outcomes between people with asymptomatic and symptomatic AF, found no difference in all-cause mortality, cardiovascular mortality, or stroke between the two groups.^30^ A cohort study of 5555 patients in UK general practice found that asymptomatic AF was associated with an increased risk of stroke and all-cause mortality compared to people without AF.^31^ Cardiac implanted devices have also been used to assess the relative risk of stroke in patients with asymptomatic, device-detected AF. In the ASSERT study over a mean 2.5-year follow-up, there was a 2.5-fold increase in the risk of stroke or systemic thromboembolism in those with episodes of asymptomatic AF compared to no AF.^25^

Asymptomatic AF therefore appears to carry an increased stroke and mortality risk compared to sinus rhythm. Whether the same level of risk will be seen in individuals who have silent AF detected as a result of screening in the general population remains to be seen.

## What burden of atrial fibrillation is associated with significant stroke risk?

Around 25% of people with AF have paroxysmal rather than sustained AF.^32^ Atrial fibrillation burden refers to the proportion of time that a patient is in AF. Although this is not included in risk prediction tools such as CHA_2_DS_2_VASc, AF burden appears to be an important factor in predicting stroke risk. A systematic review found persistent and permanent AF were associated with a greater risk of thromboembolism and all-cause mortality compared to paroxysmal AF, even when controlled for key variables such as age and sex.^33^

**Figure 2:**
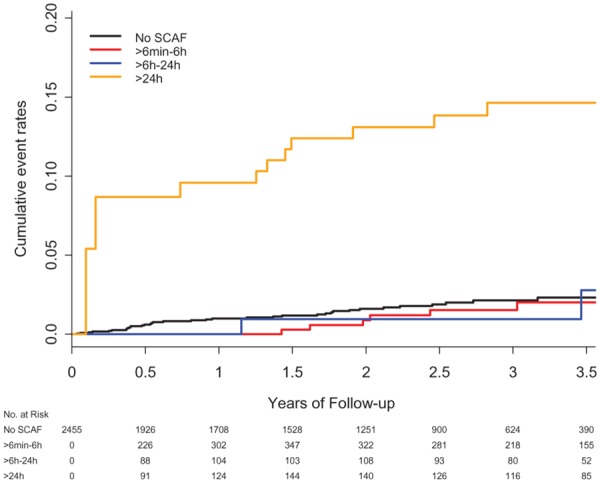
Extended Kaplan–Meier curves of ischaemic stroke/systemic embolism stratified by time-dependent durations of subclinical atrial fibrillation (SCAF). Reproduced from the Van Gelder *et al*.^21^

Extended screening using devices such as pacemakers, implantable cardiac monitors, patches, or smartphones will detect brief episodes of paroxysmal AF and atrial arrhythmia, termed atrial high rate episodes (AHRE). These episodes are frequently detected in people with implanted cardiac devices (*Table 1*). Different durations of arrhythmia have been defined to distinguish AHRE and AF from electrical artefact and the clinical significance of short episodes of arrhythmia remains uncertain. In the ASSERT study, people with asymptomatic AF of over 24 h duration had a significant increase in risk of stroke or thromboembolism compared to those without AF [adjusted hazard ratio (HR) 3.24, 95% confidence interval (CI) 1.51.6.95] but those with asymptomatic AF under 24 h duration were not found to be at increased risk (*Figure 2*).^21^

Currently, the diagnosis of AF is based on the detection of the typical arrhythmia for at least 30 s. Risk stratification by AF burden cannot be applied to patients diagnosed using a single ECG. Stroke risk scores such as CHA_2_DS_2_VASc have not been validated for AF diagnosed in the setting of extended monitoring to help determine if anticoagulation will be of net benefit. If existing guidelines were applied to AF detected by extended screening, even people with a very low burden of disease would be recommended treatment.^34^ Under these circumstances, the bleeding risk from anticoagulation may be greater than the stroke risk reduction.

It should, however, be noted that in patients in sinus rhythm with stable atherosclerotic vascular disease, rivaroxaban (2.5 mg twice daily) plus aspirin (100 mg once daily) caused a significant reduction in ischaemic stroke vs. aspirin alone, although also with an increased risk of major bleeding.^35^ This suggests that patients with a high CHA_2_DS_2_VASc score have a significant stroke risk regardless of AF and some people in sinus rhythm may benefit from anticoagulation. New stroke risk stratification scores may need to be developed that factor in both AF burden and cardiovascular risk factors for people diagnosed with AF via extended screening.

## Atrial fibrillation, silent vascular brain lesions, and the risk of dementia

Patients with AF are not only at an increased risk of overt stroke but also more likely to suffer a clinically silent vascular brain lesion. A recent study pooled the results from three different observational studies of patients undergoing ablation for treatment of AF who had no known prior history of a stroke or transient ischaemic attack (TIA).^36^ Amongst 175 participants, brain magnetic resonance imaging (MRI) before the ablation procedure detected silent ischaemic brain lesions in 14 (8%) and cerebral microbleeds in 30 (22%).^36^ The mean CHA_2_DS_2_VASc score was 1 and over two-thirds had paroxysmal AF. All received anticoagulation for at least 6 weeks prior to study assessment. Another study assessed the relationship between vascular brain lesions and cognitive function among 1737 patients with AF aged 65 years or older, 90% of whom were taking oral anticoagulation.^37^ Brain MRI detected a non-cortical infarct in 30%, microbleed in 22%, and white matter lesion in 99%.^37^ The majority of the non-cortical infarcts were in patients with no documented history of stroke or TIA and therefore classified as ‘clinically silent’. Large non-cortical infarcts were associated with decreased cognitive function. Nearly half of these patients (46%) were classified as having paroxysmal AF, with 24% permanent and 30% persistent.^37^ A recent Korean population cohort study, which included 10 435 people diagnosed with AF, also demonstrated that AF is linked to an increased risk of cognitive decline, including both vascular and Alzheimer’s dementia, even in patients with no history of a clinical stroke and after adjusting for other stroke risk factors.^38^ Anticoagulation was associated with a 39% reduction in incidence of dementia (HR 0.61, 95% CI 0.54–0.68).^38^

Clinically silent vascular lesions therefore appear to be common among patients with AF and may occur independent of whether AF is paroxysmal or persistent.^39^ Developing new strategies to help protect against cognitive decline is important given ageing populations and the growing prevalence of dementia globally. However, whether silent infarcts and the resulting vascular lesions explain the reported association between AF and dementia remains uncertain. Multiple pathways may connect the two and much existing evidence comes from relatively small, cross-sectional studies, leading to a call for further research in this area from expert consensus statements.^40^

## Would targeted screening in high-risk groups be effective?

Risk models that are based on individual characteristics, medical history, or blood biomarkers may play an important role in identifying people at sufficiently high risk for AF to warrant screening.^27^^,^^41^ Crucially, risk scores that are commonly used to predict stroke in patients with AF (e.g. CHA_2_DS_2_VASc) are also able to predict AF risk in patients in sinus rhythm, suggesting that they may be used to identify individuals who are more likely both to display AF upon screening and to benefit from treatment.^42^ Targeted screening of high-risk groups is therefore possible, potentially significantly reducing the number needed to screen.

**Figure 3:**
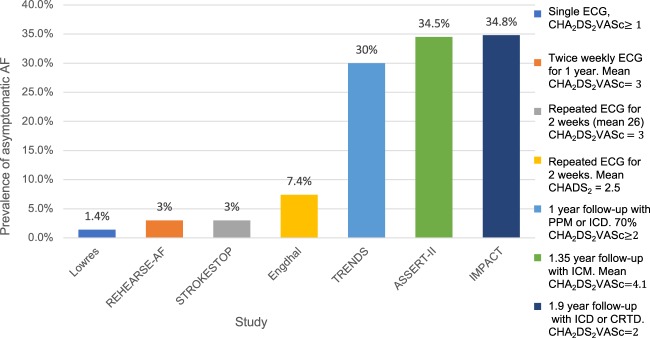
Prevalence of asymptomatic atrial fibrillation by screening method and stroke risk score.

Screening studies that have recruited enriched cohorts report varying rates of newly detected AF, depending on the method of screening and population risk factors (*Figure 3*). For example, the REHEARSE-AF study recruited 1001 patients with a mean age of 72.6 years and CHA_2_DS_2_VASc score of 3 and randomized them to twice weekly home ECG screening using the AliveCor Kardia system or usual care.^43^ In the screening group, there were 19 new cases of AF detected over 1-year follow-up, compared to 5 in the usual care arm at a cost of $10 780 per AF diagnosis. In contrast, the ASSERT-II study used implantable subcutaneous ECG monitoring to detect AF over 16-month follow-up.^27^ They recruited 256 people in sinus rhythm at baseline with a mean age of 74 years and CHA_2_DS_2_VASc score of 4.1 and found 34.4% of participants had at least one episode of AF lasting over 5 min.^27^ Short episodes of atrial arrhythmia were found in around one third of all patients across studies where an implanted cardiac device or monitor was used for extended screening in high-risk individuals (*Figure 3*).

## What would be the best approach to screening?

**Figure 4:**
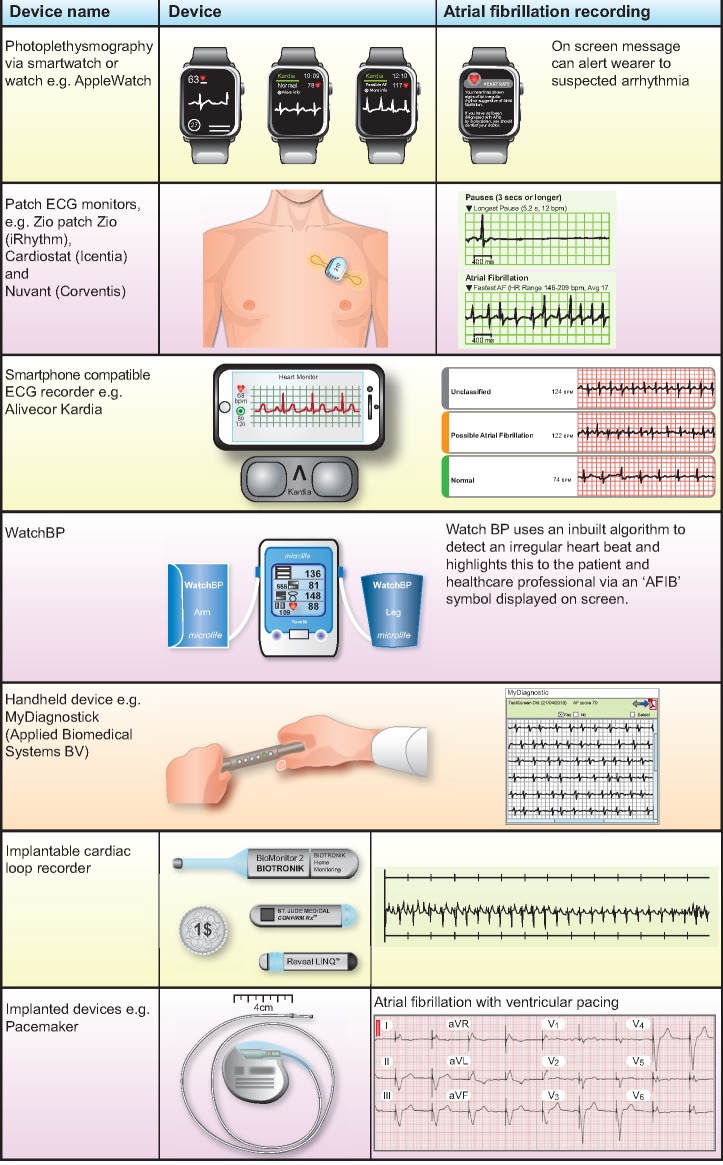
Examples of devices used to detect atrial fibrillation and how they record the arrhythmia.

A range of new technologies have been developed aimed at improving the accuracy and rates of AF detection (*Table 2*, *Figure 4*). These offer flexibility, so patients can periodically self-test at home and capture their heart rhythm at the time of symptoms. Blood pressure monitors automate pulse rhythm detection, making this another opportunity to screen for AF. Extended continuous monitoring is already recommended for detection of AF following embolic stroke of undetermined source (ESUS).^50^ Patch ECG monitors now offer a simple alternative to Holter or loop recorders to allow extended screening for AF in other settings. Smartphones, watches, and Fitbits are nearly ubiquitous and incorporate increasingly sophisticated technology to capture personalized health data. This includes photoplethysmography and related smartphone algorithms, which can be used to detect AF with reported sensitivity and specificity above 90% (*Table 2*), when compared with 12-lead ECG interpreted by a Cardiologist.^45^^,^^51^ The increased flexibility of rhythm monitoring devices has seen AF screening studies extend to home detection, pharmacy trials and traditionally difficult to reach populations, such as rural communities in resource-poor settings, with increases in the rate of AF detection throughout.^47^^,^^52^

**Take home figure:**
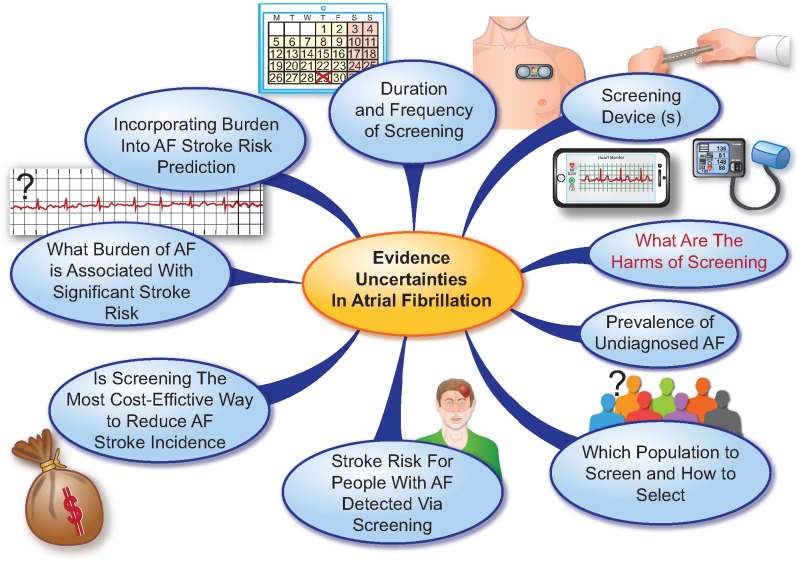
Evidence uncertainties in atrial fibrillation screening.

**Table 2:** New technology for atrial fibrillation detection

Type of technology	Example device	How it works	Advantages	Performance
Photoplethysmography via smartwatch or watch	AppleWatch Technology compatible with wide range of smartphones	Photoplethysmography creates a tachogram using intermittent blood flow monitoring. An algorithm within the device analyses this information to identify people with possible AF, who are then notified.	Provides extended home monitoring, health care provider may not need to fund device. The high level of participant engagement with the AppleWatch study suggests good acceptability.	The reported positive predictive value of the AppleWatch tachogram and notification was 71% and 84%, respectively.^44^ DETECT AF PRO report photoplethysmography algorithm sensitivity of 91.5% and specificity of 99.6% for AF detection, based on 5 min of heart rhythm analysis.^45^
Blood pressure monitor to detect AF	WatchBP Home A (Microlife) Omron M6 (Omron)	Automated BP monitors can detect variation in pulse regularity and will highlight suspected AF.	Time efficient, high sensitivity, increases likelihood of AF detection across health care settings, e.g. at health check or pharmacist review.	WatchBP had a sensitivity 95% (90/93) and specificity 86% for AF detection, when tested in 405 Cardiology patients.^46^
Handheld device or smartphone compatible ECG recorder	Kardia (Alivecor) Zenicor ECG (Zenicor) MyDiagnostic (Applied Biomedical Systems BV)	Handheld devices used to capture single ECG recording, which can be stored on the device or downloaded.	Patient controlled so enables home recordings to capture the heart rhythm at the time of symptoms. Data can be electronically transferred for review by a health care professional.	Kardia had a sensitivity of 98.5% (67/68) and specificity 91.4% (849/929) for AF diagnosis in one study screening 1000 patients aged ≥65 years.^47^
Patch ECG monitors	Zio (iRhythm) Cardiostat (Icentia) Nuvant (Corventis)	Adhesive patch, providing continuous rhythm monitoring for up to 2 weeks. Data were analysed by company software.	Offers prolonged period of non-invasive monitoring. Can be self-applied by most patients and has good acceptability.	Found to be more sensitive than 24 h Holter for AF detection.^48^ A 2-week Zio patch pilot study of 75 participants aged ≥55 years old and with ≥2 AF risk factors found new silent AF in 5.3%.^49^

Recently, the preliminary results of the AppleWatch study were presented at the 2019 American College of Cardiology annual conference.^44^ Nearly 420 000 people with no history of AF or current anticoagulation self-enrolled in the study. The AppleWatch identified people who may have AF using intermittent monitoring of a photoplethysmographic signal (*Table 2*).^44^ When the AppleWatch detected a pulse irregularity, participants were notified to contact the study doctor for a consultation to decide whether they should wear a 1 week patch ECG monitor to screen for AF. Only 0.5% of participants received a notification, though over 80% of participants were younger than 55, and notifications rose to 3% of people aged ≥65 years. Close to 30% of participants were lost to follow-up. Of the 2161 participants who received an irregular pulse notification, 450 (20.8%) eventually wore and returned an ECG patch. Of these, 153 (34%) had AF detected with a positive predictive value for the irregular pulse notification of 84%.^44^ More than 20% of the AF identified was over 24 h duration.^44^

The AppleWatch is part of a trend towards increasingly affordable and accessible technology becoming available to the public who would then be able to self-monitor their own health. The study demonstrates how wearable technology may help identify cases of AF earlier. However, thus far studies of photoplethysmography have tended to attract relatively young participants, with AF detection rates below 1%.^53^ Used in this way, the AppleWatch and similar Smartphone devices would result in many false-positive results, increasing demands on health care services despite uncertainty as to the clinical value of detecting AF in low risk individuals.

Whilst extended or more frequent screening is likely to result in increased rates of AF detection, no comparative trials have as yet been done with any of these devices. As such their relative merits, accuracy and role in diagnostic pathways remain uncertain and need to be further evaluated.

## What are the harms to patients of screening for atrial fibrillation?

A recent systematic review of screening for AF, found no eligible studies that had compared the harms of screening compared to no screening.^54^ However, a number of potential harms from screening exist. Population-level screening could lead to significant numbers of false-positive results. A range of non-invasive approaches to screening are possible, almost all with a sensitivity and specificity above 90%.^55^ This is comparable to current cancer screening approaches, such as mammography for breast cancer (sensitivity 82.3–88%, specificity 91.6–99.2%) or faecal immunochemical testing (sensitivity 79%, specificity 94%).^56^^,^^57^ However, unlike these other screening programmes where a positive screening result would prompt further more detailed investigation to confirm the diagnosis, a positive AF screening result or confirmatory ECG would directly trigger the initiation of anticoagulant treatment where appropriate.

Wearable AF detection devices have been undergoing refinements to improve their specificity still further, such as the Apple Watch addition of a single lead ECG to the existing photoplethysmography. Yet the positive predictive value of any screening test for AF would depend on the expected AF prevalence in that population as well as the sensitivity and specificity of the AF screening device. Even if the screening method used had a 95% specificity for AF diagnosis, up to 50 000 people per million screened might be falsely diagnosed.^58^ These patients might be exposed to unnecessary additional investigations, health anxiety around the implications of their diagnosis and an increased bleeding risk for those started on anticoagulation therapy. This has been demonstrated by recent anticoagulation studies in other settings, such as a RCT of rivaroxaban vs. aspirin for secondary stroke prevention in ESUS, which was terminated early due to increased rate of major haemorrhage with rivaroxaban, with no difference in recurrent ischaemic stroke rate.^59^

## Is screening the most cost-effective way to reduce stroke risk in atrial fibrillation?

Systematic opportunistic screening is thought to be more cost-effective than a systematic population screening programme, based largely on the findings of the SAFE study.^11^^,^^55^ Any national screening programme would require new country-specific management pathways to ensure coordinated treatment and follow-up. This would have significant cost implications in terms of programme infrastructure, the screening device and treatment. Funding may be better spent improving and standardizing existing AF management.^12^ International anticoagulation rates persistently fall below target levels.^60^ Amongst the 94 000 people who suffered an ischamic stroke in the Riks-Stroke registry, over 22% had previously diagnosed AF but only 16% of these had received a prescription for anticoagulation in the 6 months prior to their stroke.^6^ Higher CHA_2_DS_2_VASc score was inversely correlated with anticoagulation prescribing, meaning those at greatest risk were least likely to be prescribed treatment.^6^ The significant economic impact of suboptimal anticoagulation prescribing in high-risk populations has been demonstrated, as has the cost-effectiveness of anticoagulation for stroke prevention.^61^^,^^62^ Even if future studies demonstrate AF screening is effective at reducing stroke rates, the comparative cost-effectiveness with other initiatives to improve anticoagulation prescribing will need to be established.

## Why is further research needed and what will the planned screening trials add?

Many of the AF screening trials to date have used non-randomized, cross-sectional study designs making it impossible to compare AF detection with usual care in the same population.^19^^,^^20^^,^^46^ Approaches to screening based on intermittent or single time-point assessment will miss cases of paroxysmal AF.^63^ More recent trials, in particular, those using smartphone technology, have tended to recruit younger participants and therefore may be subject to selection bias by under-representing higher risk groups and therefore also underestimating AF prevalence.^43^^,^^44^ Reporting of important participant characteristics, such as baseline cardiovascular and stroke risk, are inconsistent across studies. Combined with the heterogeneity in screening devices and study populations, this makes it difficult to compare reported outcomes. Screening studies to date have focused primarily on AF case detection and few have gone on to assess the future care or longer-term outcomes for people with screen-detected AF.

Upcoming studies are powered to detect changes in stroke rates, major bleeding, and mortality.^14^ The results will help establish the relative risks and benefits of anticoagulation in people with screen detected AF compared to symptomatic patients. Further studies are comparing anticoagulation with apixaban or edoxaban vs. aspirin for prevention of stroke or systemic thromboembolism in people with AHRE, which will help inform which groups detected via extended monitoring may benefit from treatment based on burden of AF.^64^^,^^65^ Other screening studies are recruiting people at high risk of AF,^15^^,^^66^^,^^67^ in the hospital setting,^68^ or comparing different devices to determine which is most accurate and cost-effective.^16^^,^^66^^,^^69^ Both large scale and smaller streamlined screening studies will provide information to inform policymakers in deciding whether national AF screening programmes would be clinically and cost-effective, possible to implement at scale and how acceptable this would be to patients.

## Conclusion

Atrial fibrillation screening meets many of the Wilson and Junger^70^ criteria for a successful screening programme. Subclinical AF is a common, important and growing health problem. There are a range of potential approaches to screening, including non-invasive tests with a high degree of acceptability to patients. Atrial fibrillation is an important stroke risk factor and there is established, highly effective treatment available that can reduce this risk of thromboembolic events. Failure to identify and treat AF leaves patients at a considerably higher risk of stroke, disability and death.^70^ At present, however, the evidence does not show that screen detected AF patients have the same cardiovascular risks or benefits from anticoagulation. Nor is it certain whether screening improves health outcomes in terms of stroke-related morbidity and mortality or all-cause mortality. Treatment standards for patients with established AF remain suboptimal. Resources may be better invested in treating high-risk patients with symptomatic AF given the known benefits.

Innovations in technology are likely to mean the public becomes increasingly aware of their own health data and allow people to self-diagnose AF through smart technology. Research is needed to answer key questions such as what burden of AF is significant and what is the best risk stratification tool for determining stroke risk in this context. Large scale randomized trials, powered to endpoints including cost-effectiveness, stroke, and death can help address these evidence gaps and determine the best way to invest health care resources in AF treatment.

## Funding

N.R.J. is supported by a Wellcome Trust Doctoral Research Fellowship (203921/Z/16/Z). C.J.T. is funded through a National Institute for Health Research (NIHR) Academic Clinical Lectureship. F.D.R.H. acknowledges his part-funding from the NIHR School for Primary Care Research, the NIHR Collaboration for Leadership in Health Research and Care (CLARHC) Oxford, the NIHR Oxford Biomedical Research Centre (BRC), and the NIHR Oxford Medtech and In-Vitro Diagnostics Co-operative. L.B. acknowledges funding from the British Heart Foundation (BHF), UK Medical Research Council and the NIHR Oxford BRC. B.C. acknowledges funding from the BHF and the NIHR Oxford BRC. The views expressed are those of the authors and not necessarily those of the NHS, the BHF, the NIHR, or the Department of Health and Social Care.

**Conflict of interest:** B.C., L.B., and N.R.J. are part of the trial team for the AMALFI AF screening study, funded by the NIHR Oxford BRC and iRhythm Technology Inc. B.C. is the Principal Investigator of a BHF-funded AF screening study within UK Biobank. B.C. acknowledges non-financial support from Roche Diagnostics outside the submitted work. L.B. acknowledges financial support in the form of research grants to the University of Oxford from the British Heart Foundation, UK Medical Research Council, Merck and The Medicines Company outside the submitted work. F.D.R.H. is co-Principal Investigator on the SAFER AF Screening Trial, funded by the NIHR. C.J.T. reports speaker fees from Vifor and Novartis and non-financial support from Roche outside of the submitted work.
